# Co-designed, culturally tailored cervical screening education with migrant and refugee women in Australia: a feasibility study

**DOI:** 10.1186/s12905-022-01936-2

**Published:** 2022-08-20

**Authors:** Rosalie Power, Jane M. Ussher, Alex Hawkey, Olivia Missiakos, Janette Perz, Olayide Ogunsiji, Nikolina Zonjic, Cannas Kwok, Kate McBride, Melissa Monteiro

**Affiliations:** 1 Translational Health Research Institute, School of Medicine, Western Sydney University, Sydney, Australia; 2 Family Planning NSW, Ashfield, NSW Australia; 3 Community Migrant Resource Centre, Parramatta, Australia

**Keywords:** Cervical screening, Health promotion, Cultural tailoring, Migrant and refugee women, Qualitative

## Abstract

**Background:**

Participation of culturally and linguistically diverse (CALD) women from migrant and refugee backgrounds in cervical screening is crucial to eliminate cervical cancer as a public health problem within the next 20 years. However, CALD women report low participation in cervical screening. Barriers to participation can be addressed with culturally tailored, community-based programs. There is a need for research to explore the process, feasibility, acceptability and barriers to cultural tailoring in the delivery and evaluation of cervical screening health education.

**Methods:**

CALD community health workers took part in a 2 day training program then co-designed, culturally tailored and co-facilitated cervical screening health promotion forums within their communities. Forums were delivered to a total of seven groups, involving 12 sessions and 71 CALD women. The forums were evaluated for feasibility, acceptability, implementation and effectiveness using a survey, interviews and observations. Data were collected from CALD women, facilitators and researchers.

**Results:**

The co-design and co-delivery of cervical screening health promotion forums was time and resource intensive however allowed for deeper cultural tailoring resulting in engagement with ‘hard to reach’ CALD women, improved health literacy and intention to screen. Flexibility in the intervention implementation was crucial to ensure forums were responsive to community interests and needs. Online delivery of the forums in response to the COVID-19 pandemic was acceptable to most groups.

**Conclusions:**

Co-designed, culturally tailored cervical screening health promotion forums are feasible and acceptable to CALD women, in both face-to-face and online formats. Adjustments to the intervention protocol were recommended to improve future implementation.

## Background

Incidence and mortality from cervical cancer in Australia has dropped by more than 50% since the introduction of routine screening in 1991 [1]. However, rates remain high among culturally and linguistically diverse (CALD) women from migrant and refugee backgrounds who are less likely to engage in preventative health practices, including cervical screening and human papillomavirus (HPV) vaccination [2–7]. Disproportionate rates of screening between CALD and Australian born women is a major public health concern as women who have never engaged in cervical screening are more likely to develop cervical cancer and have poorer treatment outcomes [8]. Western Sydney, a geographic region with the largest migrant and refugee populations in New South Wales (NSW) Australia [9], and the location of the present study, reports the lowest rates of cervical screening participation in NSW [10]. Barriers to cervical cancer screening include CALD women’s perception of risk for cervical cancer [11–14]; lack of understanding of cancer and screening [12, 15–20]; embarrassment, fear or misconceptions associated with cancer screening [12, 14, 15, 21, 22]; shame associated with sexuality and sexual health [21]; impact of female genital mutilation (FGM) [23]; cultural or religious beliefs about tests, such as threats to virginity [15, 21]; fatalistic beliefs which undermine primary prevention [21]; language issues [12, 24, 25]; and mistrust related to health care engagement [26].

There is evidence that women who receive information and advice about cervical screening and early detection of cancer, which serves to increase health literacy, are more likely to attend for cancer screening [27, 28]. Health literacy facilitates informed decision making, contributing to better health outcomes and empowerment [29]. Many CALD women recognise that they have inadequate knowledge about cervical screening and HPV vaccination, and have identified that is an area of health literacy they would like to improve [30, 31]. There is a particular need for cervical screening health literacy programs given recent changes to the national cervical screening program in Australia. In 2017 the screening program changed from cytology-based screening every 2 years for women aged 18–69 years to HPV testing every 5 years for women aged 25–74 years [32]. With these changes, Australia is predicted to eliminate cervical cancer as a public health problem within the next 20 years [33]. Given nearly one-third of the Australian population were born overseas, participation of CALD women in cervical screening is crucial to achieve these targets.

Previous research has identified that health literacy and other barriers to participation in screening can be addressed with culturally tailored, community-based programs [27, 34, 35]. Cultural tailoring of interventions means “the adaptation of the study design, materials and other components of the intervention to reflect cultural needs and preferences at the population level” (Torres-Ruiz, Robinson-Ector [36], p. 3). This can include peer education [37], delivered in the woman’s own language [19], that aims to increase health literacy and allay fears [27], by incorporating women’s belief systems into explanations and communications for cervical screening [27]. Research suggests that information on cancer prevention and screening may be more acceptable for women from CALD backgrounds if it is delivered by community health workers (CHWs), assisted by health care professionals (HCPs) [19, 31, 38]. Even though literature suggests that many CALD women report a desire for information on screening [38], ideally face-to-face [39] in a group format [31, 35] they are often reluctant to attend cancer focused information sessions. There is also some evidence that CALD women are more willing to attend women’s general health information sessions that include information on cervical screening [31, 38, 40].

Previous research that has evaluated culturally tailored, community-based cervical cancer education with CALD women has used peer educators or bi-cultural community workers [27, 41–44], information materials in participants’ language [44, 45] and encouraged engagement through strategies such as culturally specific foods [46]. Interventions have often used the same strategy across multiple groups [47, 48] and have been evaluated using pre and post knowledge surveys [27, 42, 43] and screening uptake [41]. There is a need for research to explore the process, feasibility, acceptability and barriers to cultural tailoring in the delivery and evaluation of cervical screening education. This includes adaption of messages to integrate cultural values, norms and religious beliefs [49], the need to be responsive to intersectional characteristics, such as cultural background, age, migration experience, and language and literacy needs [21, 50], and the feasibility of including cervical screening as part of a more general women’s health information session. In the context of the coronavirus pandemic (COVID-19) there has also been a call for research that explores the acceptability and feasibility of delivering health promotion programs to CALD women using online formats [31], an issue of particular concern given the overall reduced screening rates within the general population during the pandemic [51].

This study evaluated the process, feasibility and effectiveness of co-designed, culturally tailored health promotion forums to address lack of understanding of changes to the Australian cervical screening program and limited health literacy around cervical screening within CALD communities. Our research questions: Are co-designed, culturally tailored cervical screening health promotion forums feasible and acceptable to CALD women? Are the health promotion forums including co-design, delivery and evaluation practical to implement, and what are the barriers to delivery? What impact do the health promotion forums have on CALD women’s intention to screen and health literacy, regarding cervical cancer and its screening?

## Methods

### Design and setting

This single arm non-randomized feasibility study was conducted in Western Sydney, Australia. This study was a collaboration between Western Sydney University and two organisations that provide state based sexual and reproductive healthcare and community support to migrants and refugees: Family Planning NSW (FPNSW) and the Community Migrant Resource Centre (CMRC). Integrated knowledge translation (IKT) underpinned the research and the collaborative relationships [52]. IKT involves co-design, collaboration and power sharing between researchers and knowledge users, to co-create knowledge and enhance the relevance and implementation of research findings [53]. In this study, researchers, community stakeholders and knowledge users collaborated to co-design and co-implement community-based cervical screening health promotion forums tailored to individual communities and groups. The study received ethical approval from the Western Sydney University Human Research Ethics Committee (H13200), and reciprocal approval from FPNSW. Informed consent was obtained from all participants and all methods were carried out in accordance with the ethical standards of these institutes and with the 1964 Helsinki declaration and its later amendments or comparable ethical standards. The study conformed to the CONSORT extension to pilot and feasibility trials guidelines, recommended for the reporting of non-randomised feasibility studies, with adaption or non-use of items that were not relevant [54].

### The intervention: co-designed and culturally tailored cervical screening health promotion forums

#### Active co-design and cultural tailoring through consultation with community stakeholders

Researchers at Western Sydney University, in collaboration with organisational partners FPNSW and the CMRC, initiated the study, as part of an ongoing program of research into CALD women’s sexual and reproductive health. After funding was obtained for the feasibility study, a meeting was held to develop and refine the research design and study population, involving the researchers, organisational partners, and key community stakeholders. This group made the decision to focus on CALD women aged 18 years and older from African or Middle Eastern backgrounds, reflecting the demographic of major CALD groups within Western Sydney, and a recognised need for cervical cancer information in these communities [55].

Following this meeting, six CALD community health workers (CHWs) were recruited through networks identified by CMRC, in order to consult with CALD women across a range of African and Middle Eastern communities. All CHWs were women who had strong relationships with their community and/or experience facilitating community health promotion activities. Each CHW organised one consultation meeting with a group of women from their community (*n* = 2–12 women per meeting) to inform the co-design and cultural tailoring of the forums. During the meetings, women discussed knowledge and beliefs about cervical screening in their communities, and suggestions for culturally acceptable and engaging methods for the forum delivery and evaluation. Ideas generated included inviting women to share food (i.e., lunch, BBQ), take part in an exercise program, offering attendance certificates, or incorporating education about cervical screening with language classes, or sessions on coping with stress or broader cancer screening or health information (including breast and bowel cancer, and bone health). Discussion about forum evaluation identified the need for methods that are suitable for women with low literacy and limited English, and that are time efficient. The meetings also served to build relationships and facilitate engagement with potential forum participants. Consultation participants were given a $30 (AUD) voucher to acknowledge their time contribution.

#### Community health worker training

CHWs participated in a 2 days training program, consisting of 10 modules, facilitated by FPNSW and Western Sydney University. The training aimed to build CHW knowledge and skills to co-design, culturally tailor and co-facilitate cervical screening information with their communities. The details of the training program are provided in Table 1. In summary, day one addressed knowledge about cervical screening; day 2 explored facilitation methods and strategies for tailoring the forums for individual communities, building on ideas and suggestions generated during the community consultations. Due to time constraints, two women who completed the training were not able to be involved in the co-design of the forums. Instead, they each recommended another woman from their community, both community leaders, who were individually trained by FPNSW.

**Table 1:** Overview of community health worker training program

*DAY ONE: Cervical screening knowledge*
Module 1: Anatomy - what is the cervix?
Module 2: The human papilloma virus (HPV) and HPV vaccinations
Module 3: Cervical screening eligibility and screening guidelines
Module 4: Cervical screening test procedure and self-collection method
Module 5: Understanding cervical screening test results
Module 6: Changes to the national cervical screening program (i.e., screening eligibility and timelines)
Module 7: Myths and barriers to cervical screening
*DAY TWO: Cervical screening forum cultural tailoring and facilitation*
Module 8: Building group rapport including discussing sensitive topics with CALD migrant and refugee communities
Module 9: Answering tricky questions about cervical screening
Module 10: Tailoring cervical screening health education for your community

#### Refining forum co-design and cultural tailoring

Previously developed CALD cervical screening health promotion materials, developed and evaluated by FPNSW and funded by the Cancer Institute NSW [56], were adapted for the present intervention. The materials informed key messages about cervical screening delivered to each group, including information about changes to the cervical screening test, screening eligibility and test procedure. The forum design and delivery of key messages were co-designed and culturally tailored with each group.

CHWs worked with a heath professional (i.e., health promotion officer or GP) and/or a health promotion trained researcher at Western Sydney University to refine the co-design and cultural tailoring of the forum, or forum series, for their community. Design considerations included forum time, duration, number of sessions, roles of the CHW and health professional (i.e., as co- or lead-facilitators) and facilitation methods. Cultural tailoring involved adapting key messages and developing content to address cultural norms and religious beliefs, knowledge gaps and misinformation.

#### Forum eligibility, invitations and recruitment

CALD women aged 18 years and older from African or Middle Eastern backgrounds and living in Western Sydney were invited to attend the women’s health forum. Invitations were circulated by CHWs, through migrant and refugee community organisations (text, WhatsApp and email lists), on social media (Facebook), and through hard copy flyers distributed to nearby venues, including shops, libraries and religious institutions and snowball sampling. The invitations, co-designed and tailored to each community and group in collaboration with CHWs, stated that cervical screening would be discussed at the forum. Invitations also included details about other information or activities that were part of the forum (e.g. lunch, Zumba class, other health screening information, children’s activities), as well as the forum logistics such as date, time and location, and the logos of any collaborating community organisations.

#### Forum delivery

Forums were delivered to a total of seven groups, involving 12 sessions and 71 CALD women between December 2019 and October 2021. A description of each forum is provided in Table 2. In summary, three forums were delivered face-to-face; two prior to the COVID-19 pandemic and one following the easing of restrictions. Face-to-face forums ran for between one and a half to four hours. The other forums were planned as face-to-face events, but changed to online events due to lockdown restrictions on social gathering. One event that was expecting over 100 participants was due to be held the day after the lockdown was announced. The event was required to be postponed and was re-organised for a later date using an online modality. The online forums were facilitated on zoom over one to three sessions ranging between 60 and 90 min per session.

**Table 2:** Overview of co-designed, culturally tailored cervical screening health promotion forums

Community	Modality	Duration (h)	Forum topics/activities	Forum attendees	Languages	Facilitation
West African (young women)	Face-to-face (weekend, daytime)	4	Cervical screening; Zumba class; lunch	7	English and Krio	Casual group discussion using flipchart co-facilitated by CHW and health professional
West African^+^^	Online (weekday, evening)	1	Stress management	14	English	Online presentation and group discussions facilitated by health professionals
		1.5	Cervical screening	25		
		1.5	Breast and bowel screening	21		
East African	Face-to-face (weekday, daytime)	4	Cervical screening; breast screening; Zumba class; lunch	10	Juba-Arabic	Presentation facilitated by CHW; health professional provided support answering participant questions
East African (young women)	Online (weekday, evening)	1	Stress management	4	English and Somali	Online presentation and group discussions co-facilitated by CHW and health professional
		1.5	Cervical screening	1		
East African	Online (weekend, evening)	1.5	Stress management; cervical screening	7	English and Arabic	Online presentation and groups discussions facilitated by health professionals
Multiethnic African group^+^	Face-to-face (weekend, daytime)	1.5	Cervical screening; breast screening	10	English	Formal presentation using ppt facilitated by health professional
Middle Eastern^+^^ (multiple countries)	Face-to-face (weekday, daytime)	1.5	Breast and bowel screening	8	English and Arabic with translator	Online ppt presentation and groups discussions facilitated by health professional
	Online (weekday, evening)	1.5	Bone and joint health	8		
		1.5	Cervical screening	8		

*CHW* community health worker

^+^Established community group; ^a single series forum for men was also hosted addressing stress management

For each face-to-face and online forum, culturally tailored information about cervical screening was provided alongside other health information requested by the community, including; breast (*n* = 4 groups) and bowel screening (*n* = 1), stress management (*n* = 3) and bone health (*n* = 1). Activities that accompanied the face-to-face forums were a Zumba exercise class (n = 2) and the sharing of food (*n* = 3). At the request of community leaders, for 2 groups the forums with women were followed by health promotion forums for men, held at a later date, covering cancer screening and mental health, and facilitated by a male researcher/community member. One CHW requested that her group be provided with gift vouchers to acknowledge women’s participation in the forums. Childcare was provided for face-to-face forums. Two forums were coordinated through established community groups, one group was delivered as part of a full day program organised by an African women’s network and the remainder were delivered to groups organised by the CHW for the specific purpose of this project. In most cases, women at the forums knew each other. As suggested by participants during community consultations, two forums were tailored to young women (i.e., 18–24 years).

CHW led the forums, with additional cervical screening information provided by a co-facilitator, a health promotion officer or GP, and information on other aspects of the forum, such as stress management, provided by one or more of the researchers. The facilitators, co-facilitators and researchers were introduced to CALD women at the beginning of the forums.

### Participants

Seventy-one CALD women attended the forums of whom 49 participated in the formal forum evaluation (i.e., survey and focus group interviews; participation rate = 69.0%). Women were eligible to take part in the evaluation if they had attended the cervical screening information session. The number of women who took part in the focus group interviews ranged from two to ten people per group. Demographics of women who completed the evaluation are provided in Table 3, women were from countries across North, East and West Africa and the Middle East. Approximately one quarter (*n* = 12, 24%) had never previously had a cervical screening test or pap smear, including women (*n* = 2) who were not yet eligible for the test due to their young age.

**Table 3:** Characteristics of evaluation participants

	Total (*n* = 49)
*Participant age*^*a*^	
18–24 years	9 (18%)
25–49 years	30 (61%)
50–74 years	9 (18%)
*Cultural background*	
West African	23 (47%)
East African	15 (31%)
Middle Eastern	5 (10%)
North African	3 (6%)
Different African or Middle Eastern background	3 (6%)
*Never previously had a CST or pap smear*	12 (24%)

^a^Missing *n* = 1

### Evaluation

The co-designed culturally tailored health promotion forums were evaluated for (1) feasibility and acceptability: focusing on CALD women’s engagement with the content and delivery of the forums [57]; (2) implementation: the practicality of the forum co-design, delivery and evaluation [57] and; (3) effectiveness: the impact of the forums on CALD women’s screening intention and health literacy [58]. Data were collected using a range of methods [59] including survey, interviews and observations, and from multiple perspectives [58], including CALD women, facilitators and researchers. Using a range of data collection methods served to ensure that data collection was optimised, responsive to the language and literacy needs of each group and forum modality (i.e., online or face-to-face).

#### Survey with CALD women

CALD women who were forum participants were invited to complete a survey assessing their knowledge (4 items; pre-intervention), fear about cervical screening (1 item, pre-intervention) and intention to have a cervical screening test in the future (1 item; repeated pre- and post-intervention). The survey questions, provided in Table 4, were derived from questions used by FPNSW for evaluation of health education sessions with CALD communities. The same survey data was collected with each group, tailored to the language and literacy needs of each group of CALD women and the forum modality. Methods included a paper survey, online multiple-choice poll, and an envelope activity in which participants in a face-to-face forum were read aloud knowledge/attitude questions and asked to put coloured cards indicating their response as true (green), false (red) or unsure (orange) into envelopes, eliminating literacy needs.

**Table 4:** Cervical screening health literacy: Knowledge, fear and screening intention

Knowledge about cervical screening	Correct answer (*n*, %)
If a woman feels well, does she still need to have a cervical screening test every 5 years?^a^	34 (81)
Does a woman only need to have a cervical screening test if she has pain or bleeding?^a^	32 (76)
If you have ever had sex, do you need to have a cervical screening test every 5 years?^a^	34 (81)
If you have only ever had one sexual partner, do you still need to have a cervical screening test every 5 years?^b^	25 (66)
Proportion of women who answered all knowledge questions correctly^b^	7 (18)

Fear about cervical screening and screening intention	Yes (n, %)	No (n, %)	Unsure (n, %)
Does thinking about the cervical screening test scare you?^a^	17 (40)	21 (50)	4 (10)
PRE: Will you have a cervical screening test in the future?^c^	32 (82)	0 (0)	7 (18)
POST: Will you have a cervical screening test in the future?^c^	36 (92)	1 (3)^d^	2 (5)

^a^*n* = 42; ^b^*n* = 38; ^c^*n* = 39; ^d^n = 1 not eligible for cervical screening due to absence of a cervix

#### Focus group discussions with CALD women

At the end of each forum or forum series, CALD women were invited to take part in a focus group interview to understand their experiences of the forum/s. Using a semi-structured interview guide CALD women were asked what they liked about the forum/s; their experience of the delivery and modality; how the forum/s could be improved; and other ways to engage women from their community in cervical screening. Focus groups were conducted in English or Arabic, with the CHW or community member acting to translate. Participating women gave verbal informed consent. Focus groups were only audio recorded in instances where CHWs felt that this would be appropriate and where practical. Detailed notes were taken by one of the researchers in instances that focus group discussions were not audio recorded.

#### Semi-structured interviews with forum facilitators

To understand experience of the IKT co-design process and delivery CHW forum facilitators and co-facilitators were invited to take part in a semi-structured interview about their perception of the forum acceptability and implementation. Interviews with forum facilitators aimed to understand experiences of the forum co-design, CALD women’s engagement with the content and delivery of the forums, and how the forums including co-design, delivery and evaluation could be improved. Interviews were conducted either over the phone or online (i.e., using videoconferencing software). All interviews were audio recorded.

#### Participant observations

Researchers who attended the groups conducted observations of the forum co-design, delivery and evaluation, to understand the intervention implementation and evaluation constraints. Researchers recorded detailed field notes about the coordination and co-design of the forums, participant attendance and engagement at the forums; facilitation methods such as strategies adopted by facilitators to culturally tailor the forum content and delivery, and; forum and evaluation logistics such as modality, timing and duration.

### Data analysis

Survey data were collated and descriptively analysed, for percentage of women agreeing to knowledge and attitude items. Focus group discussions and interviews were transcribed and then verified for accuracy by listening to the audio recording whilst reading the transcript. Transcripts were de-identified. Qualitative data was thematically analysed using guidelines described in Braun and Clarke [60]. Transcripts and observation notes were read and re-read for familiarity and to identify initial ideas for codes. A coding frame was developed through discussion and consensus between team members. This included first order and sub-codes such as ‘importance of individualised, culturally tailored sessions’, ‘need for flexibility in this project’ and ‘format of the sessions’. The transcripts and observation notes were then coded by one member of the research team. The coded data was read by three members of the research team and through a process of discussion and consensus were re-organised into themes. Themes were reviewed, linked back to the research questions, and refined. Preliminary findings were shared with research partners for comment and further refined until final themes were developed, and a final thematic map agreed upon.

## Results

**Fig. 1:**
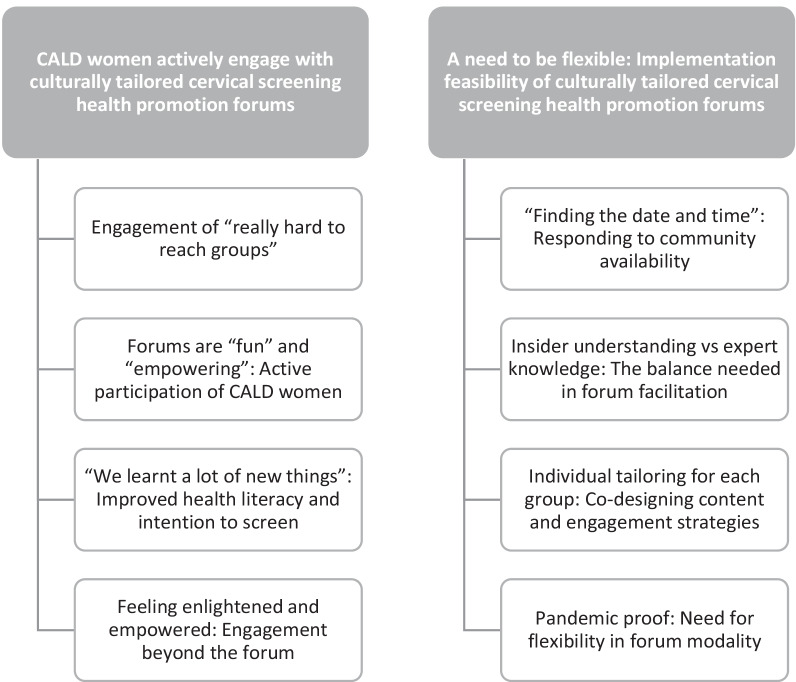
Thematic map

The descriptive survey results are reported in Table 4 and discussed alongside the qualitative evaluation findings. The results from the CALD women, the CHW, co-facilitators and observers were combined and presented under two primary themes, summarised in Fig. 1. In the results, facilitator data are identified numerically. CALD women are identified by the group they attended (e.g. West African group, multiethnic African group, etc.). Observational data is denoted descriptively (e.g. “we observed…”).

### CALD women actively engage with culturally tailored cervical screening health promotion forums

#### Engagement of “really hard to reach groups”

CALD women said they wanted to know about cervical screening, however explained that “it’s something that within our community we don’t usually talk about” [multiethnic African group] and that the topic could be “uncomfortable”, “very taboo” and “a really big deal” [West African group]. Delivery of information about cervical screening as part of a broader women’s health promotion program, including topics such as stress management and bone health, encouraged participation and engagement from women who otherwise “won’t come”, because “we don’t talk about sex” [West African group], or because they “hadn’t heard about the cervical screening before” [Middle Eastern group]. CALD women also spoke of how having activities alongside the forum such as a “Zumba session” and “great healthy food” [East African group] was an added benefit that encouraged their attendance, as “we don't have time to exercise, having this alongside the session helped” [East African group]. Attendance was also high for the face-to-face session where childcare was provided for the duration of the forum.

One co-facilitator, with many years’ experience delivering sexual and reproductive health education to CALD communities observed that using the networks of CHWs to access the community, encouraged engagement and participation by women who wouldn’t usually access mainstream community education. She shared,

I deliver this kind of content to a variety of different communities. The women that we actually targeted for this one [project], like the base level of knowledge was just surprisingly low on not just cervical screening, but a lot of things to do with their body. When we'd run the sessions [in the past], there was great improvement in knowledge…but a lot of the women had already had a cervical screening or a pap test…they weren't really the really, really hard to reach groups. [In this study] I felt like we got a sample of women that hadn't been exposed to this type of information before. Not all of them, obviously, but there was a lot of that. [Facilitator 1]

CALD women’s limited knowledge about cervical screening was confirmed by the proportion of participants who provided correct survey answers to the knowledge questions asked at the beginning of each forum. Less than one fifth (18%, *n* = 7) answered all four knowledge questions correctly. The most common misunderstanding was that women don’t require a cervical screening test if they have only ever had one sexual partner, answered correctly by 66% (n = 25) of forum participants (Table 4).

Engaging women through the networks of CHWs allowed for participation by women for whom the logistical aspects of attendance may otherwise be a barrier. CHWs for the West African and Middle Eastern groups were observed to individually communicate with women to help navigate and problem solve the logistics of joining the online forums. For example, helping women with instructions for how to download the videoconferencing software and re-sending the meeting link, avoiding drop out of women who were unable to easily log in. Similarly, the CHW of the young women’s West African group helped to connect women attending a face-to-face forum to organise and overcome issues with transport.

#### Forums are “fun” and “empowering”: active participation of CALD women

Women described the forums as “fun”, “empowering”, “effective” and “informative”. Women’s enjoyment of the forums was evident in their engagement during the sessions. Many women openly shared their experiences, often laughing, or telling jokes. They responded to facilitators in a number of ways, including engaging in open discussion during face-to-face sessions and online, using the chat function over Zoom and continually asking questions for clarification and elaboration. The “interactive” nature of the forums, including willingness and ability of facilitators to “deviate from the PowerPoint” and “let the ball roll according to what we [forum participants] wanted to know” [Multiethnic African group] was viewed by women as a “very effective way of [us] getting information” [West African group]. A high level of engagement and question asking was particularly evident in sessions that were co-facilitated by the woman GP, with forum participants asking questions about a range of health issues at the end of the cervical screening presentation. A number of participants reported appreciating the opportunity to ask questions about health topics in addition to cervical screening saying, “we could ask questions that seemed even outside [cervical screening] and still get answers” [Multiethnic African group]. This high level of engagement by participants contributed to sessions going over scheduled time, with women still willing and wanting to be present or online after the session finish time.

Facilitators were cognisant of this need for broader health information, reporting that “it was about working with what mattered to them [participants], rather than this is what we want to deliver” [Facilitator 3]. For CALD forum participants, this approach encouraged them to “share ideas within ourselves” and “develop from each other”, as “what you know someone else might not know, or someone else might know something you don’t know” [West African, young women’s group]. Facilitating the sharing of information and experiences among a group of women from similar communities was viewed very positively by CALD women, as one participant shared,

For me it was the ground opening, ground-breaking which is very good, especially when we have women’s meetings to have this kind of information distributed and discussed openly, it’s very positive and I love it. [West African group]

#### “We learnt a lot of new things”: improved health literacy and intention to screen

Participants reported that the content and delivery format of the forums resulted in them learning “a lot of new things” and that “now I'm aware to do something, you know? That’s why for me it was very inspiring” [West African, young women’s group]. One participant told us how she found the content of the forums to be accessible; “you simplify the words…it is very complicated, but then the way you've delivered the session with the simple way and the translation way, because [of this] it was very straightforward…I understood it properly” [Middle Eastern group]. Women in most groups discussed having previously received a cervical screening test. Despite this, many had not understood how cervical cancer developed, what the test was looking for and discussed a desire to learn more; “I've had a pap smear before but this was more information” [East African group]. Many participants described improving their knowledge about “about the HPV”, how it was transmitted and potentially progressed to cervical cancer, with women telling us, “the abnormal cells, that it can be treated at the beginning before it goes and causes cancer” [Multiethnic African group] and “I thought if you had cancer there, you’d have to remove the whole system. I didn’t know that different parts could have different cancers” [West African group]. This information was particularly important for those participants who were “not aware about a lot of this kind of thing” [West African, young women’s group] and facilitated women who do not yet make the screening criteria to “prepare mentally and physically for when we reach that stage” [West African group].

Cultural tailoring of cervical screening information helped address fear and concerns for women who felt they would be “too embarrassed and ashamed to go to the doctor for this” [West African group]. Fear of cervical screening was common among CALD women with just under half of women (*n* = 17, 40%) saying they felt scared when thinking about the test. Women explained feeling fearful that going for a cervical screening test could mean they already have cervical cancer, a highly stigmatised disease.

It’s the stigma around it…you know what, just going for screening, there is a perception that once you get up to go for screening then you *might* have it …so the stigma is really really…it’s terrible” [Middle Eastern group]

To address these concerns, CHWs and co-facilitators encouraged women to reflect on what stops them or other women they know from having a cervical screening test. Facilitators were also observed to spend time explaining what happens during the test (i.e., test procedure) including that the test only takes a few minutes and encouraged women to share their own experiences of the test. Participants were invited to share which clinical location they attended for their cervical screening, to address the barrier of women not wanting to have a cervical screening test with a family doctor.

When asked about their intention to have a cervical screening test in the future, 36 (92%) of women said that they intended to have the test, up from 32 (82%) at the beginning of the forums (Table 4). Only one woman said that she would not have a cervical screening test in the future, however, clarified that she was now aware that she was not eligible for the test due to absence of a cervix. For some forum participants, family responsibilities took precedence over their own health care, contributing to avoidance of the cervical screening test, something that they acknowledged needed to change; “A lot of us are avoiding it [cervical screening]. We don’t pay attention to our bodies, we focus on our families and other things, we need to focus on women’s health” [East African group]. For others, cervical screening was discussed as something that could benefit the family, “if a woman love their families, they have to attend these sessions to prevent themselves to be held from cancer, to be healthy, to be able to look after their families, their kids” [Middle Eastern group].

#### Feeling enlightened and empowered: engagement beyond the forums

Women’s engagement with the forum content extended beyond the immediate forum, with many saying that they felt enlightened and empowered to now “spread the awareness” and “pass the message around”. This included telling family and friends “you need to go to see your doctor to do this test” [West African, young women’s group], “you need to get tested regularly to prevent cancer” and that “there is a vaccine to prevent the virus, HPV” [Multiethnic African group].

I have a friend…but she hasn't gone yet.… Because you've explained everything and how important it is to get tested, I'm going just tell her: “it’s very straightforward, it’s very important to have the cervical test because you can have the abnormal cells before it goes to cause cancer. So it can be treated at the beginning, so like pre cancer, to be treated not to cause the dangerous cancer”. [Middle Eastern group]

Participants described a strong sense of social responsibility to “help other women” saying that “it's not all about us, it's about everyone” [West African, young women’s group]. Several women spoke of taking information about the cervical screening back into their communities, “for us it's more than just like learning something. It's something we can put it back into our lives and our family and our friends” [West African, young women’s group]. Another woman “running a mother’s group” said she would “take this forward and share it with the group and mums. We need this” [East African group]. The availability of information resources at many of the face-to-face forums was viewed by women as a helpful addition, allowing them to share information about cervical screening with family and friends after the forum,

If we have little book like this one, I can go home and show my mum or my sister or anyone around us. I can say, ‘Oh, this one is the cervical screening – I would like you to come it’s very important’. It’s very good for me to take this home. [West African group]

At the completion of the forums women frequently enquired “what’s next”, wanting more sessions to be held in the future and at one forum requesting the details of the GP facilitator to book a clinical consultation for further information and support. Many women said they would like to have the option to have a cervical screening test as part of the health promotion forum, “we can do it on the same day”. Women also discussed organising themselves in groups to encourage and support each other to complete the test, evident in this interaction between focus group participants.

P1: Wouldn’t it be good if we had a day where it’s like a little support circle where we all go to a GP one day, or like a few of us and we all get tested, obviously we’re not going to be in the room when each of us are doing it but it’s like my sisters accountability partner like I have –

P2: (interrupts) smear sisters (group laughs). [Multiethnic African group]

Other ideas to improve participation and engagement with cervical screening and future health promotion initiatives included using social media to share health information and promote events, delivering the forums through churches, and using community champions to “champion this cause” [Multiethnic African group]. Women suggested “there should be an awareness forum for the men” as “they know nothing about the cervical cancer” and “it will make them more responsible” as “it’s [HPV] sexually transmitted” [Multiethnic African group]. Additional forums addressing other aspects of women’s reproductive and sexual health were also requested, such as sessions about contraception, pregnancy and menstruation, and other topics including domestic and family violence.

### A need to be flexible: implementation feasibility of culturally tailored cervical screening health promotion forums

#### “Finding the date and time”: responding to community availability

Being responsive to communities required flexibility on the part of the facilitators in timing and duration of forums; “not every community may be available nine to five” but “that doesn’t mean that we can still not reach out to them” [Facilitator 3]. Finding a date and time that maximised the opportunity for attendance for each group, “when we thought there would be more participants” [Facilitator 2] was observed to be complex to coordinate, as it required multiple communications between the facilitators, researchers and venue (for face-to-face forums). Confirming that the forum did not conflict with other community events, such as the funeral of a community member, was also important to facilitate attendance. Some groups met during the weekday whilst children were at school, others on mid-week evenings after work and one group on Sunday evenings, to fit around religious and family responsibilities. This flexibility meant in most instances “we were able to get a very good turnout” [Facilitator 3]. The length and number of forums were determined by each group; some preferred multiple consecutive but shorter forums, others preferred a single, longer forum allowing participants to commit for a one-off timeframe.

Being responsive to community availability also required flexibility on the day of the forum, with one forum starting approximately 2 hours late due to waiting for participants to arrive and many running beyond the advertised end time. This flexibility was positioned as being key to the success of the forums, as one co-facilitator shared,

On the day there was a hundred percent effort in making sure that day worked and if it went longer than expected, it was fine. If people didn't show up and you have to wait an hour or more, it was fine…I think if the team didn't take that approach, they wouldn't have gotten the outcomes that they got, because for that reason, we were able to capture women that would run late to things because they have to drop off their kids, women that have multiple children, multiple responsibilities, women that are working that don't have time for consultations. [Facilitator 1]

#### Insider understanding vs expert knowledge: the balance needed in forum facilitation

Another requirement for flexibility was being responsive to community preferences for the roles of the CHW and co-facilitators. For some groups, facilitation was led by a health professional, irrespective of cultural background, considered by women a “reliable source” who “made the whole thing solid, ‘cause they are professionals, that is their area. So you are inclined to listen to them, than a layman, you know” [Mixed African group]. For other groups, community led facilitation meant that the sessions could be delivered in women’s languages, avoiding the need for translators and allowing for insider understanding, sensitivity and the tailoring of content to directly relate to participants. Co-facilitator 3 explained, “I was able to cite specific African-informed content and words because in the community, that's the way we relate with each other and there are specific examples that we could draw from. You know, mentioning of countries, mentioning of experience of being migrants, which I have.” Community led facilitation meant that there was a possibility of error in the delivery of messages, however the presence of the expert co-facilitator could address this:

It was heavily reliant on me as a facilitator being able to pick up what the other facilitator was saying, because there were times that she dropped a key message or said something that was actually conflicting and I needed to step in. The forums can be as community led as you want, but there needs to be an element of control for what messaging is being delivered, especially in communities that are so value driven. Giving some of that wrong information can completely put off women from actually getting the test done. [Facilitator 1]

Uncertainty about the accuracy of information was observed to be a challenge when information was delivered in a language other than English, as “you don’t actually know what is being said” [Facilitator 1].

#### Individual tailoring for each group: co-designing content and engagement strategies

Taking time to “listen to” and “understand each group” and “the barriers they face”, including cultural and religious beliefs and norms, enabled CHW and co-facilitators to tailor the delivery of cervical screening information. This included addressing misinformation and community specific myths or stigma with each group. A co-facilitator explained,

The [cervical screening] messages are going to stay the same - get tested this often, you can book an appointment this way, etc. But the real kind of intricacy and success to the project is if you can embed smaller cultural messages within to be like, okay this is relevant to you. [Facilitator 1]

For example, “for one of the East African groups, fertility was a barrier… there was so much fear around diagnoses and potentially not being able to get treatment” where as “the Middle Eastern group, it was a lot more around modesty and a lot more that linked to sexual activity, especially if the woman wasn't married” [Facilitator 1]. Due to the interactive nature of the forums, it was observed that providing individualised content for each group required experienced facilitators, who were able to talk across all aspects of cervical screening and also answer women’s questions about reproductive and sexual health more broadly.

Individual tailoring also meant that for some groups, cervical screening information was provided alongside other information about women’s health within the same session, whereas in other groups it was during a consecutive session. Co-facilitators held differing views about the approach, with one saying it was “beneficial” to “get them [CALD women] under one roof and deliver as much, you know, health-related information as possible that they are willing to spend their time on”. The co-facilitator said “the number of attendees” evidenced the success of this approach [Facilitator 3]. However, another co-facilitator acknowledged limitations to the amount of content that was reasonable to deliver in one session, “when we deliver any session to CALD group of people, do not overload them with information. I think [for one group] we had mental health part and then cervical screening, breast screening. So what I found that it might be a bit overwhelming for them” [Facilitator 2]. This could also be “tiring” for the facilitator.

While the process of co-design to tailor the forum content and delivery was crucial to the project, there were some challenges with the process. Limited availability of both the CHWs and co-facilitators meant that coordinating mutually suitable times to work on the forum design was observed to be difficult; the fine details of forums were often only confirmed close to the forum date, limiting opportunity to practise delivery. Co-facilitators also reported that the forum co-design was, “a lot more involved than I anticipated… a lot more support was required than I initially thought”. The same co-facilitator said that “there was an element of women falling into what they already know” and “stick[ing] to what was safe”, repeating similar approaches to each other. The co-facilitator acknowledged that despite this, “it worked…[CALD women] were still interested in those activities” however suggested that “more training” for the CHWs may have been beneficial as it “would allow for that creativity” during the co-design [Facilitator 1].

#### Pandemic proof: need for flexibility in forum modality

The delivery of cervical screening health promotion forums during a worldwide pandemic required flexibility of forum modality, prompting a shift from face-to-face facilitation to online part way through the study. CALD women were observed to respond positively to both modalities; women attending face-to-face said they enjoyed “seeing everyone in person” [West African young women’s group], “seeing your [facilitators] faces” and that it allowed for “more explanation and more traction” [Middle Eastern group]. Women attending online said “sitting at home and getting information” made it “easy” [West African group] to attend and that “everything was explained through the screen which was just like face-to-face” [Middle Eastern group]. However, for some women receiving information online felt to be “without feeling, there’s no emotions, no feelings on the device” [Middle Eastern group].

For co-facilitators, the different modalities each had advantages and challenges. For most co-facilitators it was their first time delivering cervical screening information online, “it was my first time online … I've never done it online before” [Facilitator 2]. Despite being “a bit nervous” [Facilitator 2] about the new mode of delivery, “online worked a lot better than I thought” [Facilitator 1], viewed as preferable for managing larger groups of participants, “there was one [forum] in the high 20 s, which is great. If we had run that in person, it wouldn't have worked. Like it would be just really, really difficult to manage 25 women” allowing “more reach and reach also in terms of women that might not have been able to attend”. However overall, the face-to-face forums were considered “more engaging” as it was easier to “change communication style and body language” to be responsive to participants, such as if “I can see they're getting bored” or “they're very curious” [Facilitator 2]. The interpersonal dynamics of the face-to-face forums enabled “more of that natural discussion” and for facilitators to “dig a bit deeper” [Facilitator 1] such as when encouraging discussion about barriers to cervical screening. A facilitator explained,

With the young women’s [West African] group, with the information about contraception that was coming up and just their understanding about their menstrual cycle and even just their reproductive system and what they actually have in their body. Like, I think maybe that wouldn't have come out if we had done that online. I mean it could have. But I think some of that in depth, really rich discussion, that is more likely to happen face to face [Facilitator 1]

Face-to-face delivery also allowed for tactile elements such as “having the speculum for women to have a look” which “worked well and was passed around during the discussion” [Facilitator 2], and information pamphlets for participants to take home, written in participant’s language.

Developing group rapport and achieving open discussion with women in some online groups was challenging, particularly among younger women, or in instances when women didn’t already know each other, “we didn't get that same rapport and we didn't get to delve into some issues as much” [Facilitator 1]. A co-facilitator explained that in one forum,

Quite a number of them [CALD women] had their cameras off, which then meant I couldn't see their body language. I couldn't see, you know, how they felt or whether they giggled or something. Even though occasionally we had some women in and out of the camera [Facilitator 3]

Co-facilitators suggested that the hesitancy of some women to have their cameras on or take part in online discussions may be because “sometimes people can be shy” and “sometimes you could see kids in the background running around” [Facilitator 1]. Online forums were observed to be susceptible to technological difficulties such as challenges logging in online and unstable internet connections resulting in participants dropping in and out of the session. Despite these challenges, co-facilitators acknowledged that “even if women weren't able to be 100 percent concentrated on every bit of detail, that was okay. We still, I felt like got some information to them” [Facilitator 1], considered a “success”.

## Discussion

The present study evaluated the feasibility, acceptability and effectiveness of co-designed and culturally tailored cervical screening health promotion forums to address changes to the Australian cervical screening program and limited health literacy about cervical screening within CALD communities. Our findings demonstrated that the forums were feasible, acceptable and effective, evidenced by CALD women’s attendance and engagement with the content and delivery of the forums, improved health literacy and intention to screen, and intention to share what they had learned with family members and peers. However, there were challenges to the implementation of the intervention. Recommendations to improve the intervention for sustained implementation, and factors to be considered for a broader study systematically evaluating this form of intervention, are discussed.

Our findings extend previous research exploring cultural tailoring in community-based cervical screening education with CALD women. By working collaboratively with CHWs to design and deliver the forums we were able to be responsive to community interests and needs, in order to achieve deeper more effective means of cultural tailoring [49]. This required flexibility across all parts of the intervention and resulted in dynamic programs and content that integrated cultural values and addressed barriers, including misinformation, myths and concerns about cervical screening unique to each group. Our approach acknowledged diversity among CALD communities and avoided a ‘one size fits all’ [31] approach that conflated cultural groups as being homogenous. Attendance at the forums was destigmatised by providing cervical screening information alongside other women’s health information and activities [40]. This strategy contributed to our successful engagement with ‘hard to reach’ women who may otherwise report lower participation in cervical screening, such as CALD women who were unmarried [61], older women [62], those with limited English [2], low community connectivity/ social support (i.e., not involved in community organisations) [63], or who have limited cervical screening knowledge [2, 5]. Despite successful engagement of CALD women, the development of individual forum programs for each community and group was time consuming and labour intensive, a potential barrier to wider implementation of the intervention. Future research and intervention programs should consider ways in which this same level of flexibility can be provided, in order to achieve effective cultural tailoring for communities and groups, within a sustainable model.

In combination, the quantitative and qualitative findings of this study suggested that co-delivered cervical screening health education shared between CHWs and health professionals can be effective in improving CALD women’s health literacy and intention to screen. However, we experienced challenges with the health forum facilitation. Whilst we allocated time after the CHW training for the forum co-design and preparation, facilitation practise between the CHW and co-facilitator often did not occur, due to time constraints. We also found that most CHWs delivered forums once only, limiting opportunity to build capacity in facilitation. This placed pressure on co-facilitators to pre-empt and manage challenges in facilitation, including ensuring the accuracy of information delivered. Previous research using peer educators or bi-cultural community workers to deliver cervical screening education with CALD women has included opportunities for CHWs to develop and practise facilitation skills [27] such as through training based role plays [64, 65] or delivery of pilot sessions [43]. We recommend that future implementation of the intervention includes additional training duration to allow the CHW and co-facilitators to practise facilitation. Our findings also emphasise the need for funded models of health promotion that enable community and sexual health organisations to train and employ CHWs in ongoing roles to enable sustained delivery of co-designed, culturally tailored cervical screening education with CALD communities and groups.

Due to the unprecedented circumstances of the COVID-19 pandemic, the present study needed to pivot to provide cervical screening health promotion using online videoconferencing. This is a novel way to provide cervical screening health promotion with CALD women [31]. Online delivery required fewer logistical components (i.e., no requirement for venue, catering, etc.) making coordination simpler, however, necessitated unique facilitation skills such as building group rapport and encouragement of participation in the absence of non-verbal cues [66]. Online facilitation skills are recommended for inclusion in our CHW training program as part of future implementation.

This study demonstrated that online delivery of cervical screening information is feasible. Online delivery of the forums also appeared to be acceptable to CALD women, evidenced in part by high attendance rates, with the exception of a forum for young East African women, which engaged only one participant. The low attendance in this one forum may be attributed to several factors that have implications for the co-design and delivery of future interventions. Firstly, although delivered as part of a forum series (a session on stress management was held the week prior with four attendees) cervical screening was the sole topic of focus for the second session. Discomfort or perceived irrelevance of the topic may have dissuaded attendance [31, 38, 40]. Secondly, although the sequencing of content mirrored a previous forum series, which engaged 25 members of a community organisation of West African women, the same strength in relationships with East African women was not evident. The West African forum was with a pre-existing group, suggesting that established groups where women are familiar with one another may facilitate participation in addressing sensitive health topics. Another possible contributor to the low attendance may be that the East African young women’s forum occurred later in the pandemic, a time when many people were anecdotally experiencing ‘zoom fatigue’ [67]. The numerous interrelated factors that contribute to the acceptability of online forums for CALD women is an area that needs future research, in order to understand barriers and facilitators to engagement in online health promotion with CALD women.

The majority of CALD women who attended our forums had previously had a cervical screening test. Despite high rates of reported screening, the forums identified gaps in women’s knowledge including limited understanding about the purpose of the test, similar to previous research [68]. This finding raised concerns about the effectiveness of practitioners to provide individual patient education when performing cervical screening with CALD women and is an important rights issue as CALD women are entitled to accessible health information to be able to give informed consent [69]. Women who have good rapport [68] with and receive information and reassurance from their health provider report being more comfortable receiving a cervical screening test [68]. Our forums help to address this problem, by providing group-based education to supplement women’s knowledge improving CALD women’s cervical screening health literacy. However, moving forward, women called for the cervical screening forums to be provided alongside active support to engage in screening. This request is similar to previous literature calling for interventions that include knowledge and behavioural health promotion [31].

### Strengths and limitations

The present study evaluated the feasibility of all aspects of the intervention, including the feasibility of evaluation methods; we experienced technical and theoretical limitations. Firstly, we found that administration of our survey took too long to complete, requiring a shorter version of the survey to be derived for following forums. Secondly, online forums experienced technical issues for attendees accessing the videoconferencing platform on mobile phones. Some CALD women were unable to view the online survey resulting in missing data. However, qualitative methods of data collection such as focus group interviews and observational data provided rich insight and understanding and did not experience technical limitations. Finally, although we observed an increase in the number of CALD women who intended to have a cervical screening test following the forums, our sample was not large enough to test statistical significance. Screening intention may be a more useful indicator of forum effectiveness in research with larger samples and if accompanied by longer term follow up to understand if screening intention was translated into screening behaviour.

## Conclusions

Our findings demonstrate that co-designed, culturally tailored cervical screening health promotion forums are feasible and acceptable to CALD women, in both face-to-face and online format. Challenges with implementation that need to be addressed in future research include the time and resource intensive nature of the development and delivery of individual forum programs for each community and group, as well as the training demands on community workers. However, our approach of co-design and co-delivery allowed for deeper, more effective cultural tailoring resulting in the engagement of ‘hard to reach’ CALD women, which has the potential to improve health literacy and intention to screen. Adjustments to the intervention protocol are recommended to improve future implementation, including increasing the duration of training for CHWs to include opportunities to practice the forum facilitation and to develop skills such as those required for online facilitation; offering dedicated roles for CHWs to enable sustained delivery of the forums, and; providing cervical screening education alongside active support for CALD women to engage in screening. Longer term follow-up is required to understand if women’s screening intention translated into screening behaviour.

## Data Availability

The datasets generated and/or analysed during the current study are not publicly available due to containing sensitive and potentially identifying patient information but are available from the corresponding author on reasonable request**.**

## Abbreviations

CALD: Culturally and linguistically diverse
CHWs: Community health workers
CMRC: Community Migrant Resource Centre
COVID-19: Coronavirus disease
FGM: Female genital mutilation
FPNSW: Family Planning NSW
HPV: Human papillomavirus
IKT: Integrated knowledge translation
